# Quantitative Analysis of Selected Microorganisms Present at Various Sites in a Prosthetics Clinic and Dental Laboratory during Complete Denture Fabrication

**DOI:** 10.3390/ijerph17103345

**Published:** 2020-05-12

**Authors:** Krystle L. Moodley, C. Peter Owen, Mrudula Patel

**Affiliations:** 1 Department of Prosthodontics, School of Oral Health Science, Faculty of Health Sciences, University of the Witwatersrand, Johannesburg 2193, South Africa; krystle.moodley@wits.ac.za; 2 Faculty of Health Sciences, University of the Witwatersrand, Johannesburg 2193, South Africa; 3 Clinical Microbiology and Infectious Diseases, School of Pathology, Faculty of Health Sciences, University of the Witwatersrand, Johannesburg 2193, South Africa; Mrudula.patel@wits.ac.za

**Keywords:** denture, contamination, Streptococci, *S. aureus*, AGNB, Candida

## Abstract

*Background*: Contamination with oral commensals and pathogenic microorganisms, and cross contamination between clinic and laboratory can occur. The amount of contamination has not been determined. *Methods*: Samples from different clinical and laboratory stages before and after disinfection (17 sites, 10 samples per stage) were collected. Laboratory surfaces and equipment were swabbed for 10 days (11 sites). Swabs were cultured for total mixed flora, Streptococci, Lactobacilli, *Staphylococcus aureus*, aerobic Gram-negative bacteria (AGNB) and *Candida*. Knowledge of infection control among staff and students was assessed. *Results*: Clinic: In total, 30–40% of the samples overall were contaminated with mixed flora and Streptococci of >100 cfu/swab; >100 cfu of AGNB and *Candida* were present on 6% and 1% of samples; 2% contained <100 cfu of *S. aureus*. Laboratory: In total, 17–48% of the samples overall were contaminated with mixed flora and Streptococci of >100 cfu/swab; >100 cfu of AGNB were present on 11% of samples; none contained >100 cfu of *Candida*. Disinfection significantly reduced the level of all organisms. Knowledge of infection control was sufficient, but compliance was poor. *Conclusion*: Although the count of mixed flora was high, potential pathogens such as *S. aureus* and *Candida* were low. In immunocompromised patients, this can become a problem.

## 1. Introduction

Denture fabrication is a multi-step process involving both clinical and laboratory procedures. Cross contamination can occur at any stage of this process because dentures are reservoirs of both opportunistic and pathogenic microorganisms [1]. Microorganisms can be transferred to the laboratory in cases where there is inadequate disinfection, putting patients, clinicians and dental laboratory staff at risk.

Proper infection control strategies must therefore be implemented to prevent disease transmission by disrupting one or more links in the chain of possible infection [2]. The Centres for Disease Control (2003) [3] has recommended that dental prostheses, impressions and other prosthodontic materials be thoroughly cleaned, disinfected with an “Environmental Protection Agency (EPA)-registered hospital disinfectant with tuberculocidal claim” and subsequently rinsed properly. The most beneficial time to disinfect is shortly after removal from the patient’s mouth to ensure that drying of blood and other bioburden does not occur. The CDC further recommended the implementation of policies and procedures for containing, transporting and handling contaminated instruments as well as continuous training for all dental health care personnel.

Studies have reported that both oral and non-oral pathogens associated with local and systemic disease are present on both contaminated prostheses and dental laboratory equipment [4,5,6]. The most prevalent contaminants were found to be bacteria such as *Bacillus species*, *Streptococcus species*, *Micrococcus species* and coagulase-negative Staphylococci. These were present in bases with occlusal rims, try-in bases and dentures sent to the laboratory, and new dentures [6]. Egusa et al. [7] also found that 8% of the impressions and 11% of gypsum cast samples were contaminated with *Candida species*. In addition, studies have also found dentures to be contaminated with bacteria from Enterobacteriaceae family (gut flora) including Klebsiella, Pseudomonas and Acinetobacter [4,5,8].

These studies suggest that some of these bacteria can cause systemic infections, particularly in immunocompromised patients. However, only qualitative tests were performed in these studies and the mere presence of bacteria does not indicate their role in the development of infections. The fundamental principles in the development of any infection are the number of pathogens and their virulence, as well as host immunity.

Quantitative analysis can reveal the true level of specific contaminants at various sites of denture fabrication. There are few studies that have described the level of contamination. Kahn et al. [9] showed that dentures can be contaminated with > 5 × 10^5^ of total bacteria per millilitre of wash. Similar results were found by Williams et al. [10], and Agostinho et al. [11] reported 10^7^ of total bacteria per millilitre of denture wash and 10^9^ of total bacteria per millilitre of polishing cone wash. Nevertheless, no studies have shown the quantities of different types of bacteria present in the prosthetic clinic during denture fabrication.

The aim of the present study was to assess the level (quantities) of contamination with selected commonly occurring microorganisms in a student prosthetic clinic during denture fabrication and at various sites in the dental laboratory. In addition, the in vitro and in vivo efficacy of disinfectants and knowledge of infection control among students, staff and laboratory personal were also assessed.

## 2. Materials and Methods

### 2.1. Study Population and Sample Size

**Figure 1:**
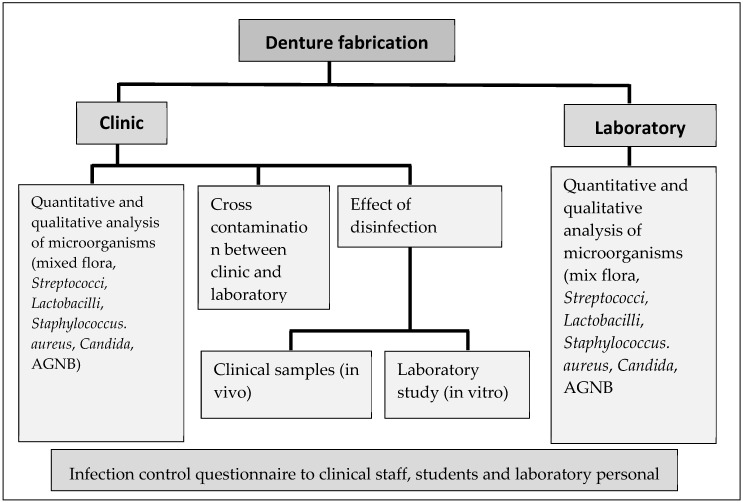
Flow diagram of the study design. AGNB: aerobic Gram-negative bacteria.

**Figure 2:**
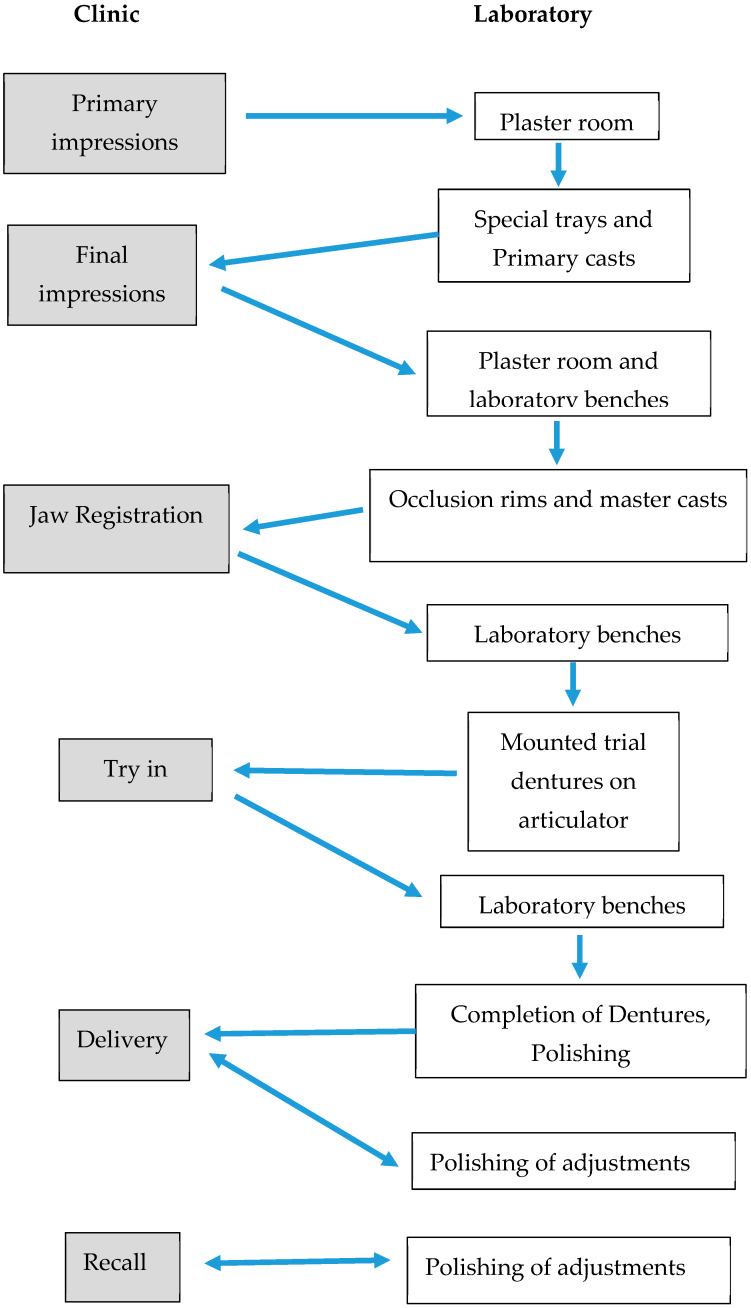
A flowchart representing the denture fabrication process.

Ethical approval was obtained from the Human Research Ethics Committee of the institution (clearance certificate M170604). Patients who attended the clinic were asked to provide informed consent for swabbing of each stage for the fabrication of new complete dentures. Patients who had existing oral pathologies were not included in this study. Due to multiple reasons, although 16 patients participated in this study, only 5 patients were followed through from the commencement of fabrication to the end stage of denture construction. The study design is given in Figure 1 and the different stages of denture production are shown in Figure 2.

### 2.2. Collection of Samples for Microbiology

Samples from denture debris, impressions, trial bases, primary and master casts and articulators were collected with sterile cotton-tipped swabs after completion of each procedure prior to disinfection (if carried out). At least 10 samples were collected for each stage of denture manufacture. Similarly, laboratory surfaces and equipment were swabbed during a 10-day period. In the laboratory, the following were swabbed: the impression collection area, the two pumice brushes, the pumice (which was not changed for each use), the grinding wheel and the working surface of each laboratory technician.

Only some students performed disinfection procedures at different clinical stages, and therefore disinfected samples were separated from the samples that were not disinfected. To establish the quantities of microorganisms at different stages of denture fabrication and in the laboratory regardless of origin, samples without disinfection were used. Disinfected material was swabbed before and after disinfection to establish the in vivo efficacy of disinfectants. Samples were processed in the Oral Microbiology laboratory within an hour of collection under normal laboratory aseptic procedures.

### 2.3. Microbiological Analysis

Microbiological analysis was performed using a technique described by Matthews and Patel [12] to isolate and identify common oral aerobic bacteria and *Candida* species. Briefly, swabs were cultured onto blood agar (BA) for bacterial count, Mitis Salivarius agar (MSA) for total Streptococci, Rogosa agar (RA) for Lactobacilli, Baird Parker media (BP) for presumptive *Staphylococcus aureus*, MacConkey agar for aerobic Gram-negative bacteria (AGNB) and Sabouraud agar (SAB) for *Candida species*. MSA and RA were incubated at 37 °C for 48 h under CO_2_, whereas BA, BP, MacConkey and SAB were incubated at 37 °C for 48 h aerobically. On BA, all the colonies were graded as 1–10 colonies, 11–100 colonies and 101 and above. Similarly, blue colonies on MSA, black colonies with halo on BP (*Staphylococcus aureus*), white colonies on RA (Lactobacilli), pink colonies on MacConkey (AGNB) and creamy colonies on SAB (*Candida species*) were read and recorded. Streptococci and Lactobacilli were not further identified to the species level. Confirmatory tests for the *Staphylococcus aureus* were not performed, and therefore *S. aureus* counts can be considered as presumptive. The results were descriptively analysed using both quantitative and qualitative analyses.

### 2.4. In Vitro Disinfectant Study

At the institution, Germicide (Germicide 3, Germiphene, Canada) is used in the prosthetic clinic to disinfect material at various stages of denture fabrication. It is a surface disinfectant, with the active ingredients being alkyl dimethyl ethylbenzyl ammonium chloride, benzalkonium chloride, and isopropyl alcohol.

The test microorganisms Candida *albicans* ATCC 90027 and *S. aureus* ATCC25923, and clinical isolates of *S. mutans* and Lactobacilli, were obtained from the Oral Microbiology laboratory. For each experiment, fresh subcultures were used. In the in vitro study, for *Candida,* Sabouraud agar was used; for Lactobacilli, Rogosa agar was used; and for *S. mutans* and *S. aureus*, blood agar was used [13]. The microbiological analysis was performed as described in the previous section.

### 2.5. The Efficacy of Disinfectants—In Vitro Study

The antimicrobial activity of Germicide was determined using the disc diffusion test [14]. Cultures were suspended in phosphate-buffered saline to obtain an optical density of 0.2 and spread onto appropriate agar plates using a swab. Optical density was measured at 405 nm using a spectrophotometer. Paper discs (3 mm) impregnated with test disinfectant were placed onto seeded agar plates with even spacing. Plates with *S. aureus* were incubated aerobically at 37 °C for 24 h. Plates with *S. mutans* and Lactobacilli were incubated under CO_2_ at 37 °C for 48 h. Plates with Candida were incubated aerobically at 37 °C for 48 h. Water as a negative control was also included. For the comparison and as a positive control, 0.2% chlorhexidine gluconate (CHX) was also included. These experiments were repeated 10 times. A zone of inhibition was measured (an average of two readings per zone) to determine the efficacy of disinfectants.

### 2.6. Knowledge of Infection Control Among Students, Staff and Laboratory Personal

To assess the knowledge and awareness of infection control, a self-generated questionnaire (Supplementary Materials) was given to the students, staff and prosthetic laboratory personal. Results were described as the percentage of types of response.

### 2.7. Statistical Analysis

Data analysis was carried out using SAS (version 9.4 for Windows, SAS Institute Inc, Cary, NC, USA). The 5% significance level was used. In vitro study results were analysed using a quantitative analysis. A two-way Analysis of Variance (ANOVA) was used to analyse results. The microbiological results were descriptively analysed using both quantitative and qualitative analyses. The quantitative results helped establish the level of contamination and the qualitative study helped establish the presence/absence of contamination. The presence and absence of contamination was determined by a Chi-square test.

## 3. Results

### 3.1. Clinical Study

**Figure 3:**
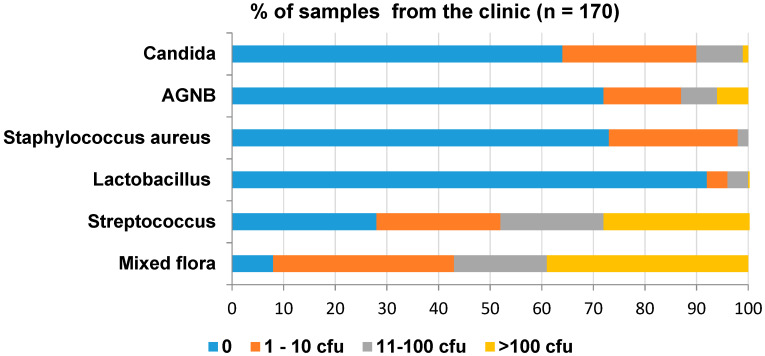
Overall level of contamination in the clinic during denture fabrication.

The overall level of contamination is shown in Figure 3. The most prevalent contaminant was Streptococci and the least was Lactobacilli. Although Candida was present in 36% of the samples, the quantities were low (1% of samples with >100 cfu/swab).

The microbiology results of the individual clinical samples are shown in Table S1 in the Supplementary Materials. More than 50% of the samples collected from primary impressions, final casts after jaw registration, bases for jaw registration in the clinic, trial bases in the clinic, and the final denture before and after polishing were contaminated with more than 100 cfu of mixed flora and Streptococci. The majority of the samples did not contain Lactobacilli. None of the samples contained >100 cfu of *S. aureus*; however, 10–20% of the primary casts from the laboratory, bases for jaw registration in the clinic and articulators from the laboratory were contaminated with 11–100 cfu. In total, 10% to 30% of the samples from primary impressions, bases for jaw registration from the laboratory, bases for jaw registration in the clinic, trial bases from the laboratory and the final denture before polishing were contaminated with >100 cfu of AGNB. Only 10% of the samples from the bases for jaw registrations from the laboratory and the final denture before polishing were contaminated with >100 cfu of *Candida*.

### 3.2. Dental Laboratory Study

**Figure 4:**
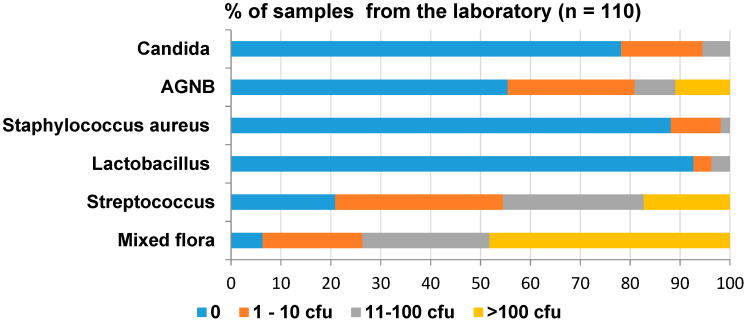
Overall level of contamination in the dental laboratory.

The results of the overall contamination in the dental laboratory are shown in Figure 4. Streptococci was the most prevalent organism. Sixteen percent of the samples contained *Candida*—of which, only 5% contained 11 to 100 cfu/swab. However, 11% of the samples contained >100 cfu/swab of AGNB. 

Detailed microbiological results of the laboratory study are shown in Table S2 of the Supplementary Materials. The least amount of Lactobacilli was found in the dental laboratory. The plaster room bench used for impressions was heavily contaminated with mixed flora. In addition, it contained *S. aureus* and AGNB. The pumice areas and the brushes were also heavily contaminated with mixed flora and contained Lactobacilli, AGNB and *Candida*. The grinding wheel was contaminated with mixed flora and mostly AGNB. Ten percent of the dental laboratory benches were contaminated with *S. aureus*, AGNB and *Candida* of up to 100 cfu.

### 3.3. Contamination to and from the Laboratory

In the work received from the laboratory (88%), mixed flora were the most prevalent and Lactobacilli were absent (Table 1). With regards to the samples going to the laboratory, all the microorganisms were present, with mixed flora being most prevalent (79%) and Lactobacilli least prevalent (10%). A significant difference existed only between the presence/absence of Streptococci (*p* = 0.045) and Lactobacilli (*p* = 0.025). The amount of the rest of the organisms was the same at both clinic and laboratory with no significant differences.

### 3.4. In Vitro Study—The Efficacy of Disinfectants

**Figure 5:**
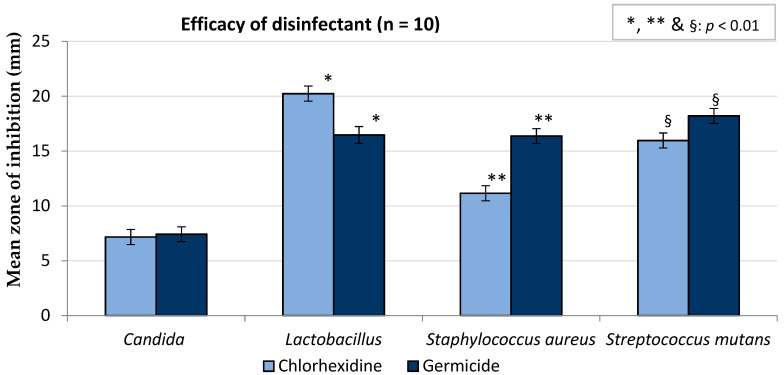
Antimicrobial activities of chlorhexidine gluconate and Germicide (error bars represent confidence intervals).

Results of the in vitro study on CHX and Germicide showed that the zones of inhibition were similar for *C. albicans* (*p* > 0.99), whereas CHX performed better than Germicide for lactobacilli (*p* < 0.01). The mean zone of inhibition of Germicide was greater with *S. aureus* and *S. mutans* compared with CHX (Figure 5). Overall, the mean zone of inhibition of Germicide (14.6 ± 0.4 mm) was significantly higher than CHX (13.6 ± 0.3 mm), with a *p* value of 0.002.

### 3.5. In Vivo Study—The Efficacy of Disinfection

In order to compare the presence or absence of microorganisms before and after disinfection, all six sites (where disinfection occurred) of testing were considered together, because of the low sample sizes. The results are shown in Table 2. “Before disinfection” data for 10 patients was available. However not all the clinicians/students disinfected material at each stage of denture fabrication, and therefore the number of samples “after disinfection” is different at each stage. There were significant reductions in the numbers of samples containing all the organisms, with *p* values ranging from 0.04 to <0.01 (Table 2). For each organism, when the samples before and after disinfection were compared individually, Germicide significantly reduced the number of samples with all the test microorganisms. The percentage reduction in samples with organisms was >72% and up to 95%.

**Figure 6:**
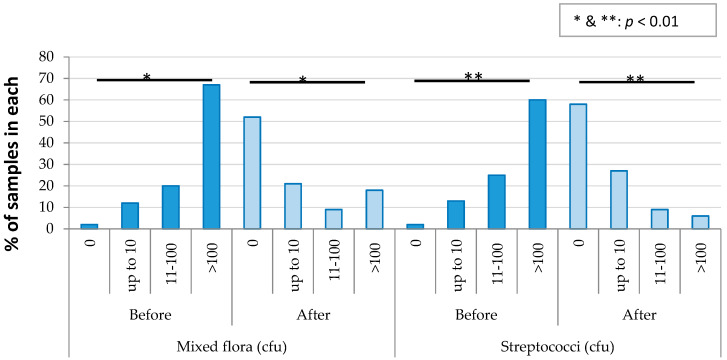
Reduction in the number of organisms due to disinfection in the prosthetic clinic.

The results of the mixed flora and Streptococci were further analysed to assess the reduction in the number of organisms due to the disinfection. The results are shown in Figure 6. The number of other organisms was not compared due to the small sample size after disinfection. Disinfection significantly reduced the number of mixed flora and Streptococci.

### 3.6. Knowledge of Infection Control Among Students, Staff and Laboratory Personal

All the clinical staff members and students knew the importance of infection control in prosthodontics and they were aware of the potential transmission of Hepatitis B and Tuberculosis. Further, the majority of them were vaccinated with Hepatitis B vaccine. Although only 60–90% washed hands before and after patient examination, all wore gloves during that time. Although they all rinsed impressions with water before sending it to the laboratory, only 87% disinfected them. However, they all knew that rinsing with water was not sufficient. Only 62% of staff members and 73% of the students were aware of the prosthodontic manual containing disinfection procedures. Both staff (68%) and students (62%) had not had training on disinfection.

The laboratory staff (*n* = 11) also expressed the importance of infection control (100%) and felt that there was a need to improve infection control procedures including disinfection (91%). However, less than 30% used gloves, masks and eye protection during laboratory procedures and did not disinfect polishing rags and wheels, or incoming dental work.

The detailed results are given in the Supplementary Materials.

## 4. Discussion

A limitation of this study is that the bacterial counts were not precise per sample site. In order to obtain a count per sample, it has to be submerged into fluid and this was not possible due to the nature of the samples and surfaces. Therefore, it was decided to thoroughly swab the sites and express counts as a category. Only one person collected all the swabs. Selected microorganisms were cultured in this study because they are common and can be considered indicator microorganisms. The amount of these microorganisms can indicate the level of contamination and possible presence of other organisms.

Although studies have shown the presence of many different types of bacteria on dentures and equipment in the clinic and dental laboratory, it is important to determine the quantities of these contaminants because mere presence is not enough to cause infections. It is important to assess the type and number of bacteria and their pathogenicity. This study has shown that although there was a high level of mixed flora and Streptococci, the level of selected potential pathogenic bacteria, *S. aureus*, AGNB and *Candida* was relatively low in the clinic during denture fabrication and in the dental laboratory.

Blood agar was used because it supports the growth of many different types of bacteria, so that mixed flora represents all of the possible microorganisms that were assessed (Streptococci, Lactobacilli, AGNB, *Candida* and *S. aureus*) as well as other organisms that were not isolated. This enables an assessment of the level of overall contamination. Streptococci have been found at all sites in the mouth and constitute a large portion of the resident oral microflora [13]. In healthy individuals, there is 100% carriage of Streptococci [12]. Therefore, in this study, it would be expected to find these microorganisms at most sites in high quantities. Lactobacilli, on the other hand, constitute 1% of the total cultivable oral microflora in a healthy individual [13]. Thus, this organism should be the least prevalent or absent at most sites in this study.

Staphylococci are not commonly isolated from the oral cavity and are considered transient, but studies have reported oral cavity carriage of up to 36% [11,12]. Sattar et al. [15] reported using *S. aureus* as a test organism, as it is considered a common resident in transient skin microflora, is often associated with a plethora of infections in humans, can survive well on contaminated hands, and is able to withstand drying, heat and some disinfectants [16]. It has also found to be the most prevalent microorganism associated with nosocomial pneumonia and surgical infections [17]. In the oral cavity, *S. aureus* has been found to be associated with angular cheilitis, parotitis and mucositis [18]. The present study showed that although 27% of the samples from the clinic carried *S. aureus*, only 2% of the samples contained 1 to 100 cfu of *S. aureus*. These were mainly from the primary casts, the bases for jaw registration, and the articulators from the laboratory. The primary casts could have been contaminated from the impressions, and in turn have contaminated the bases. These organisms were found on laboratory surfaces such as the bench where the impressions were poured: they contained >100 cfu/swab of *S. aureus*. The articulators could have been contaminated at any stage in which they were used. After the jaw registration procedure, when the casts were mounted on the articulator, 50% of the occlusion rim bases were contaminated with *S. aureus*.

Aerobic Gram-negative bacteria (AGNB) are usually common in the intestine, and 17% of healthy individuals carry these in their oral cavity. This can indicate that personal hygiene is poor or that the patient is immunocompromised or has an underlying disease [12]. Very little has been reported on the presence of AGNB in denture wearers. In this study, all the sites sampled during denture fabrication as well as the sites in the dental laboratory, contained AGNB reaching counts of >100 cfu/swab. This suggests that either contamination is constantly occurring or AGNB can survive on those surfaces. These bacteria can cause urinary tract, soft tissue and systemic infections if they pass host immune barriers. Infections in the oral cavity due to AGNB are not known, perhaps because oral mucosa does not contain receptors for the adhesins of AGNB [19]. *Candida* can become pathogenic and can cause infection, especially in immunocompromised patients [20]. The incidence of candidiasis is more common in the elderly; this has been attributed to denture hygiene as well as physiological changes in the oral mucosa, and malnutrition [13]. Denture penetration with *Candida* can be a problem subsequent to active infection. Glass et al. [1,21] reported that *Candida* penetration into acrylic had been reported as early as in the 1980s. Latib et al. [22] found that the depth of penetration of *C. albicans* increased as the length (days) of exposure increased. In addition, these cells which had penetrated the denture surface remained viable and were not affected by denture surface disinfection.

In this study, although all the sites sampled during denture fabrication contained *Candida*, only two sites reached counts of >100 —the bases for jaw registration retuned from the laboratory, and the final denture before polishing. However, the dental laboratory was contaminated with *Candida*, with four places reaching counts of <100 cfu. *Candida* can survive for longer time periods than oral bacteria. Transmission can occur via hands and other vehicles such as porous (e.g., cloth) and non-porous (e.g., articulators) items [20]. *Candida* are commensals which are frequently encountered in the mouths of healthy individuals. The prevalence of *Candida* carriage can vary in healthy individuals (35–55%), denture wearers (74%) and cancer patients with prostheses (84%) [13,23]. In a commensal state, 29% of healthy individuals can carry more than 1000 cfu/mL without showing an overt infection [24]. Mathews and Patel (2018) [12] have also reported a mean cfu/mL of 719 for *Candida* in the oral cavities of healthy individuals.

In this clinic, the results also showed that although the staff and students are aware of the importance of infection control, they were not following the procedures, because only a fraction of samples were disinfected (Table 2) even though it was evident that the disinfectant used in the clinic was adequate. However, it was also evident that there was a need for a strict protocol for both the clinic and the laboratory, as well as regular oversight for conformity to such a protocol. Dentures are reservoirs of pathogenic and opportunistic pathogens and can be a vehicle for cross contamination between patients and dental personnel [2]. Therefore, dental prostheses, impressions and other prosthetic materials should be thoroughly disinfected with an EPA-registered hospital disinfectant with tuberculocidal claim and subsequently rinsed properly. In addition, regular testing of the efficacy of whatever disinfectants are recommended in any protocol is essential, as some products will be more effective than others. If used regularly at each step of denture fabrication, proper disinfection (or even cold sterilisation [25]) would reduce cross contamination. This has to be carried out in partnership with the dental laboratory staff because the results also showed that cross contamination occurred from the laboratory to the clinic and vice versa. Furthermore, effective communication between the laboratory and clinic personnel is necessary to enable proper disinfection procedures and ensure that materials are not damaged or distorted by possible disinfectant overexposure and in order to prevent duplication of disinfection procedures. The results of the survey are therefore of concern, since knowledge of need was not transferred into practice, and imply that, apart from strict protocols and oversight, continuous training for students, clinical and laboratory staff is clearly necessary, as also stated by the CDC guidelines [2].

## 5. Conclusions

This study has shown that the number of bacteria varies with the stage of the denture fabrication process and the type of bacteria. There appears to be considerable cross contamination during denture fabrication between the clinic and the dental laboratory. Although the amount of mixed flora, which also generally consist of harmless environmental bacteria (Bacillus), was high, the amount of potential pathogens such as *S. aureus* and *Candida* was low. In normal healthy individuals, this may not pose a greater risk of contracting infections but this can become a problem in immunocompromised patients. Adequate disinfection can minimize the risk of contamination. Therefore clinicians, clinic staff and laboratory personal should receive regular infection control education and all stages of denture manufacture should be disinfected before leaving the clinic/office.

## Figures and Tables

**Table 1:** The overall amount of contamination that came from and went to the laboratory during the stages of denture construction.

Microorganism	% Samples with Microorganisms		*p*-Value
	From Laboratory (*n* = 80)	To Laboratory (*n* = 100)	
Mixed flora	88	79	0.17
Streptococci	54	69	0.045
Lactobacilli	0	10	0.025
*Staphylococcus aureus*	26	19	0.28
AGNB	26	21	0.48
*Candida*	33	26	0.41

**Table 2:** Effect of disinfection in the prosthetic clinic during the denture fabrication process.

Site	Disinfection (*n*)	No of Samples with Organisms					
		Mix Flora	Streptococcus	Lactobacilli	*S. aureus*	AGNB	*Candida*
Primary impression	Before (10)	10	10	4	4	8	3
	After (7)	3	2	0	0	2	1
Final impression	Before (10)	10	10	2	2	1	2
	After (6)	1	1	1	0	1	0
Bases in clinic	Before (10)	10	10	3	5	5	5
	After (5)	3	3	1	0	0	0
Trial bases in clinic	Before (10)	10	10	2	5	2	5
	After (3)	2	1	0	0	0	0
Final denture before polishing with pumice	Before (10)	10	10	3	3	3	6
	After (7)	3	5	0	0	2	0
Final denture after polishing with pumice	Before (10)	10	10	0	3	3	8
	After (5)	4	2	0	1	0	1
Overall results	Before (60)	60	60	14	22	22	29
	After (33)	16	14	2	1	5	2
Reduction in samples with organisms (%)	73	77	86	95	77	93	
Comparison of before and after disinfection *p* value	Per organism	<0.01	<0.01	0.04	<0.01	0.03	<0.01
	Overall	<0.01					

## Supplementary Materials

The following are available online at https://www.mdpi.com/1660-4601/17/10/3345/s1, Table S1: Level of microbial contamination in the dental clinic during denture fabrication, Table S2: Level of microbial contamination in the dental laboratory.
