# Supplementary material for: Quantitative Analysis of Selected Microorganisms Present at Various Sites in a Prosthetics Clinic and Dental Laboratory during Complete Denture Fabrication
Source: Int J Environ Res Public Health. 2020 May 12;17(10):3345. doi: 10.3390/ijerph17103345 (PMC7277632; doi:10.3390/ijerph17103345)
Supplement: Supplementary file 1 [file ijerph-17-03345-s001.pdf]

Supplementary Materials: Quantitative Analysis of Selected Microorganisms Present at Various Sites in A Prosthetics Clinic and Dental Laboratory During Complete Denture Fabrication

Questionnaire: Clinicians and students

Academic status: **Staff** ☐ **Postgraduate student** ☐ **Undergraduate student** ☐

Questions: Please circle your answer

1 Is infection control important while performing Prosthodontic treatment? Yes No Don't know

2 Have you been vaccinated for Hepatitis B? Yes No Don't know

3 Can Hepatitis B be transmitted through saliva? Yes No Don't know

4 Can Tuberculosis be transmitted through saliva? Yes No Don't know

5 Can HIV be transmitted through saliva? Yes No Don't know

6 Do you wash your hands before and after patient examination? Yes No Sometimes

7 If "yes" or "sometimes":

What do you wash your hands with? ……………………………………………………………………………………..

…….

8 Do you use gloves during prosthodontic treatment? Yes No Sometimes

9 Do you rinse impressions with water before sending to the lab? Yes No Sometimes

10 Do you disinfect your impressions before sending to the lab? Yes No Sometimes

If "yes" or "sometimes":

a. What type of disinfectant is it? ………………………………………………….. OR: Don't know

b. What is the trade name? ………………………………………………………………. OR: Don't know

c. What is its composition (chemical contents)?

………………………………………………………………………………………………. OR: Don't know

11 Do you just rinse the trial bases, dentures etc. with tap water? Yes No Sometimes

12 Is rinsing with tap water sufficient to remove contaminants? Yes No Don't know

13 When you send a procedure to the laboratory, do you inform them if you have disinfected or not? Yes No Sometimes

14 Does the lab inform you if their work has been disinfected? Yes No Sometimes

15 Have you received training on how to disinfect at each stage of the denture fabrication process? Yes No

16 Are you aware of the infection control procedures indicated in "The Pros Book"? Yes No

17 Do you feel the infection control procedures you use are adequate? Yes No Don't Know

18 Are you aware of the infection control procedures used in the dental laboratory? Yes No

19 Do you feel the laboratory infection control procedures are adequate? Yes No Don't know

20 Please feel free to add any other comment:

Questionnaire – Dental laboratory staff

Questions: Please circle your answer

1 Does the laboratory have an infection control (disinfection protocol) policy for work coming from the clinics? Yes No Don't know

2 Can infections be transmitted through dental procedures? Yes No Don't know

3 Is infection control important in dentistry? Yes No Don't know

4 Have you been vaccinated for Hepatitis B? Yes No Don't know

5 Can Hepatitis B be transmitted through saliva? Yes No Don't know

6 Can Tuberculosis (TB) be transmitted through saliva? Yes No Don't know

7 Can HIV be transmitted through saliva? Yes No Don't know

8 Is infection control important while performing dental laboratory procedures? Yes No Don't know

9 Do you wear gloves when working in the laboratory? Yes No Sometimes

10 Do you wear a mask when working in the laboratory? Yes No Sometimes

If "Yes" or "sometimes", for what procedures? ……………………………………………………………………….

…….

11 Do you wear eye protection when working in the laboratory? Yes No Sometimes

If "Yes" or "sometimes":, for what procedures? ……………………………………………………………………..

…….

12 Do you feel the need for your work surfaces to be disinfected? Yes No Don't know

13 Do you in fact disinfect your laboratory surfaces? Yes No Sometimes

If "Yes" or "sometimes": give the name of disinfectant you use:

…… OR: Don't know

14 Are you informed if the work received from the clinic has been disinfected? Yes No Sometimes

15 Do you disinfect work received from the clinic? Yes No Sometimes

If Yes or "sometimes", how do you disinfect:

(a) Alginate impressions: ………………………………………………………………………………………………………..

(b) Rubber- base impressions: ……………………………………………………………………………………………….

(c) Denture bases (Jaw reg, trial bases etc.): ………………………………………………………………………..

16 Do you disinfect work you send back to the clinic? Yes No Sometimes

(a) If Yes or "sometimes", how:

………..

17 Do you disinfect rag wheels, burs, brushes before polishing? Yes No Sometimes

(a) If Yes or "sometimes", how:

………..

18 Do you feel the laboratory infection control procedures are adequate? Yes No Don't know

19 Do you inform the clinic if the work you send out has been disinfected? Yes No Sometimes

20 Do you use fresh pumice each time you polish a denture? Yes No Sometimes

21 Please feel free to add any other comment:

Table 1. Level of microbial contamination in the dental clinic during denture fabrication.

Site	n	Quantity cfu/swab	Mixed flora	% samples with organisms				
				Streptococci	Lactobacilli	*S. aureus*	AGNB	Candida
Primary impression before disinfection	10	0	0	0	60	60	20	70
		1-10	0	10	0	40	20	20
		11-100	20	20	30	0	30	10
		>100	80	70	10	0	30	0
Primary cast from lab	10	0	0	30	100	60	30	30
		1-10	50	30	0	30	60	60
		11-100	20	30	0	10	10	10
		>100	30	10	0	0	0	0
Final impression before disinfection	10	0	10	10	80	80	90	80
		1-10	40	40	20	20	10	20
		11-100	20	20	0	0	0	0
		>100	30	30	0	0	0	0
Final cast after jaw registration	10	0	0	30	100	90	80	80
		1-10	50	30	0	10	10	20
		11-100	0	20	0	0	10	0
		>100	50	20	0	0	0	0
Bases for jaw registration from lab	10	0	50	60	100	100	90	90
		1-10	30	30	0	0	0	0
		11-100	20	0	0	0	0	0
		>100	0	10	0	0	10	10
Bases for jaw registration clinic before disinfection	10	0	0	0	70	50	50	50
		1-10	0	0	20	30	10	40
		11-100	30	40	10	20	20	10
		>100	70	60	0	0	20	0
Articulator from lab	10	0	10	30	100	50	70	60
		1-10	50	60	0	40	30	40
		11-100	30	10	0	10	0	0
		>100	10	0	0	0	0	0
Trial bases from lab	10	0	10	70	100	70	70	70
		1-10	60	10	0	30	10	0
		11-100	10	10	0	0	10	30
		>100	20	10	0	0	10	0
Final cast from lab	10	0	10	30	100	70	90	70
		1-10	40	30	0	30	10	20
		11-100	20	30	0	0	0	10
		>100	30	10	0	0	0	0
Trial bases in clinic before disinfection	10	0	0	0	80	50	80	50
		1-10	10	10	10	50	20	40
		11-100	40	40	10	0	0	10
		>100	50	50	0	0	0	0
Final cast after try in	10	0	0	30	100	80	80	50
		1-10	50	40	0	20	10	30
		11-100	20	20	0	0	10	20
		>100	30	10	0	0	0	0
Articulator in clinic after try in	10	0	30	40	100	80	80	90
		1-10	50	30	0	20	10	10
		11-100	20	20	0	0	10	0
		>100	0	10	0	0	0	0

Final denture from lab	10	0	10	60	100	80	80	70
		1-10	40	10	0	20	10	30
		11-100	20	20	0	0	10	0
		>100	30	10	0	0	0	0
Remount cast from lab	10	0	0	60	100	90	80	90
		1-10	50	10	0	10	20	10
		11-100	20	10	0	0	0	0
		>100	30	20	0	0	0	0
Final denture before polishing with pumice and before disinfection	10	0	0	0	70	70	70	40
		1-10	0	0	10	30	0	50
		11-100	0	0	10	0	0	0
		>100	100	100	10	0	30	10
Remount cast clinic	10	0	10	20	100	90	90	80
		1-10	50	40	0	10	10	10
		11-100	0	20	0	0	0	10
		>100	40	20	0	0	0	0
Final denture after polishing with pumice before disinfection	10	0	0	0	100	70	70	20
		1-10	20	20	0	30	20	40
		11-100	10	30	0	0	10	40
		>100	70	50	0	0	0	0

Table S2. Level of microbial contamination in the dental laboratory.

Site	n	Quantity cfu/swab	Mix flora	% samples with organisms Streptococci	Lactobacilli	*S. aureus*	AGNB	Candida
Plaster room bench for impressions	10	0	10	0	90	90	50	100
		1-10	10	20	0	10	30	0
		11-100	50	70	10	0	20	0
		>100	30	10	0	0	0	0
Pumice area 1	10	0	0	0	50	100	0	40
		1-10	0	0	30	0	20	40
		11-100	0	40	20	0	20	20
		>100	100	60	0	0	60	0
Pumice brush 1	10	0	0	10	100	100	60	90
		1-10	10	20	0	0	20	10
		11-100	10	50	0	0	20	0
		>100	80	20	0	0	0	0
Pumice area 2	10	0	0	0	80	100	20	70
		1-10	0	20	10	0	20	20
		11-100	0	20	10	0	10	10
		>100	100	60	0	0	50	0
Pumice brush 2	10	0	0	50	100	100	80	100
		1-10	30	30	0	0	10	0
		11-100	30	10	0	0	0	0
		>100	40	10	0	0	10	0
Grinding wheel	10	0	50	70	100	90	70	100
		1-10	20	30	0	10	20	0
		11-100	0	0	0	0	10	0
		>100	30	0	0	0	0	0
Clinical bench 1	10	0	0	30	100	70	40	80
		1-10	40	20	0	20	60	10
		11-100	20	40	0	10	0	10
		>100	40	10	0	0	0	0
Clinical bench 2	10	0	0	0	100	80	80	80
		1-10	20	70	0	20	20	10
		11-100	60	20	0	0	0	10
		>100	20	10	0	0	0	0
Clinical bench 3	10	0	0	10	100	80	70	60
		1-10	30	60	0	10	20	30
		11-100	40	30	0	10	10	10
		>100	30	0	0	0	0	0
Clinical bench 4	10	0	0	30	100	80	80	80
		1-10	40	50	0	20	20	20
		11-100	40	20	0	0	0	0
		>100	20	0	0	0	0	0
Clinical bench 5	10	0	10	30	100	80	60	60
		1-10	20	50	0	20	40	40
		11-100	40	10	0	0	0	0
		>100	30	10	0	0	0	0
